# The geographic and topical landscape of medical education research

**DOI:** 10.1186/s12909-019-1639-2

**Published:** 2019-06-06

**Authors:** Marshall P. Thomas

**Affiliations:** University of Global Health Equity, Kigali Heights, Plot 772, KG 7 Ave, Floor 5, Kigali, Rwanda

**Keywords:** Medical education research, Diversity, Income disparities, Low income countries, Research productivity, Research geography

## Abstract

**Background:**

Whether medical education research (MER) is primarily conducted in wealthy countries (in the “Realm of the Rich”) is the subject of an ongoing debate. Previous studies of the geography of MER publication output have relied upon proprietary databases, have not compared MER with other fields of study, and have not studied the relationship between authorship geography and topics of study. This study was designed to evaluate the geographic distribution of MER authorship and to relate this to the topics studied in MER.

**Methods:**

Authors’ countries of affiliation were identified from PubMed records by parsing and cleaning the text of affiliations and submitting them to the google maps geocoding API. The geography of publication output in MER was compared to other fields using the chi-square goodness-of-fit test. Country income classifications and medical subject heading (MeSH) terms were used to evaluate the topical contributions of countries at different income levels, and simulation was used to compute significance of MeSH term enrichment in MER papers from low income and lower middle income countries.

**Results:**

The vast majority of MER papers were contributed by authors based in high income countries. The top four countries were the United States, the United Kingdom, Canada, and Australia, with listed author affiliations in 80% of all MER papers. This percentage was greater in MER than in several other categories, including Biological Science Disciplines (48%), Medicine (69%) and Education (74%), which is a parent category of MER. Authors from low income countries contributed significantly to the topical diversity of MER. MeSH terms associated with government, community health, and health delivery were enriched in papers from low income countries, while terms associated with specialty and clinical training, technology in teaching, and professional obligations (such as workload, burnout, and empathy) were enriched in papers from high income countries.

**Conclusions:**

Geographic disparities in publication output are greater in MER than in any other field examined. The historical origins of MER in North America might explain disproportionate publication output by authors from this region. This study suggests that the MER field benefits from research contributed by authors from low income countries, and also points to potential gaps in MER (and medical education as a whole) in the developing world.

## Background

Medical education research (MER) is a comparatively young field. Historians trace its origins to the United States in the 1950s [1]. By recognizing MER as a serious field of academic inquiry, and founding journals that specialized in MER, Western medical schools and medical associations promoted its development. MER has evolved and grown considerably since its inception, but the highest impact journals in the field are still based in North America and Western Europe. Although authors from wealthy countries contribute disproportionately to research output in all fields of science, there is an ongoing debate as to whether MER, in particular, is primarily conducted in the “Realm of the Rich” [2, 3].

Previous attempts to characterize the geography of MER authorship have been limited by the tools and datasets available. Up to the end of 2014, PubMed only listed affiliations of first authors [3, 4]. PubMed papers have expert-tagged hierarchical medical subject heading (MeSH) terms that can be used to specifically filter search results by field of study. However, PubMed records themselves have not been used in previous studies of the geography of MER productivity. Instead, the GoPubMed semantic search engine [5] has been used for prior work [2, 6]. GoPubMed matches papers with *similar text* to papers that bear a given MeSH term, but it does not limit search results *only* to papers tagged with a given MeSH term or its subheadings. Though it is a powerful tool for search, a large percentage of GoPubMed search results for the “Education, Medical” MeSH term are actually papers from other fields that do not bear this specific MeSH term. As a result, it is difficult to conclude that these previous studies were, in fact, drawing from a body of literature representative of the MER field.

Recently, an analysis of author gender, imputed from the first names of authors, was used to reveal gender disparities in biology research [7]. This demonstrated that systematic computational analyses can be deployed to study questions of diversity in research output and authorship. The current study aims to use publication data to characterize the geography of MER authorship, particularly as it relates to other fields of biomedical research and the research questions pursued in MER.

## Methods

### Data collection and geocoding

PubMed records written in English with a publication date from January 1, 2015 to December 31, 2016 containing each of the following MeSH terms: “Education, Medical”, “Biological Science Disciplines”, “Medicine”, and “Education” were retrieved and downloaded in XML format on April 17, 2018. Author affiliations were parsed, split, and trimmed using regular expressions. To reduce false positives, short affiliation strings were screened for geographic keywords. Each affiliation string was submitted to the google maps geocoding API [8], and the country of affiliation was extracted from the results. If more than two matches, or no matches, were returned by the geocoding API, strings were split further and resubmitted to attempt to find a single country match. The World Bank’s income classification, which is based upon per capita income tiers [9], was used to map countries to income status. The rworldmap package in R [10] was used to generate a map showing ranked output by country.

### Statistical analyses

To compare MER with other topics of research (Table 1), 2500 publications (more than 2% of the total number of publications for each term) were randomly sampled from search results in each of three related fields. The frequency of authorship affiliation (by income status and by country) was tested with the chi-square goodness-of-fit test, comparing expected values (percentage in the MER population) with values observed in each sample. The Bonferroni correction for multiple hypothesis testing was applied. For the analysis of MeSH term enrichment (Fig. 2), a *P-*value was estimated by simulating 10,000 random samples of the same size. For clarity and readability, country names were not included in the word clouds shown in Fig. 2. The *diverse* package was used to calculate Gini-Simpson diversity of countries of affiliation. All analyses were conducted in R.

## Results

Of all of the papers matching the MeSH term “Education, Medical”, 87.2% listed at least one author affiliation, and in 98.2% of these papers at least one country of affiliation was identified. 11,947 MER papers were analyzed, representing 50,853 total authors. 145 different countries were represented in the final analysis. After cleaning and splitting affiliations into strings, 22,659 unique affiliations were identified. To validate the method, a random sample of 250 MER papers was put through the geolocation pipeline and the identified countries of affiliation were checked manually. There was a false discovery rate of 1.0% and a sensitivity of 97.0% in this sample.

**Fig. 1:**
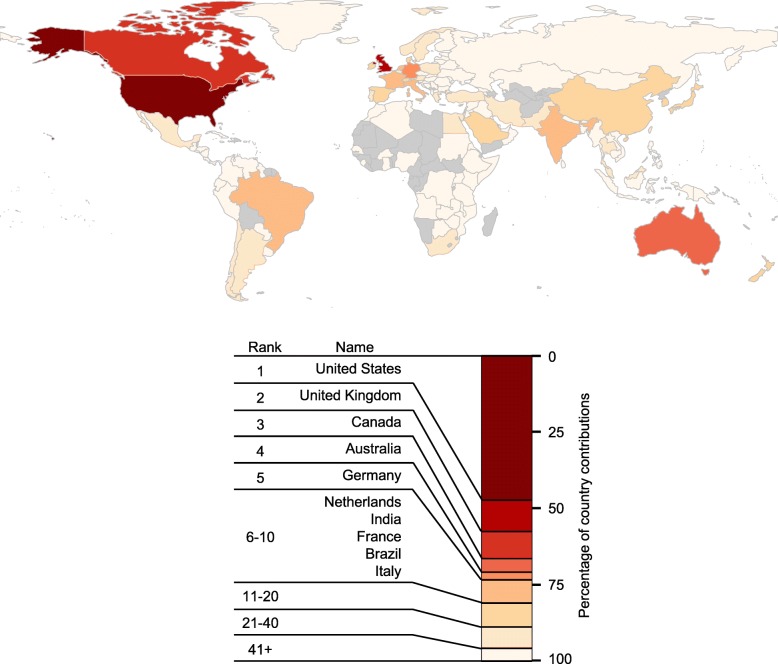
The Global Landscape of Medical Education Research Authorship Country contributions were tabulated for each paper, aggregated across all papers, and ranked by total number. Country contribution ranks are shown on the map, and the percentage of total country contributions by ranked country are shown below the map. The map was generated by the rworldmap package [10], with a base map generated from public domain data. Countries shown in grey had no identified authorships in the dataset. Unique country contributions were only counted once per paper (due to papers with authors from several countries, there are more total country contributions than papers). The vast majority of MER authors are from the US, Canada, Australia, and Western Europe

To evaluate the geography of MER research output, unique countries of affiliation (hereafter referred to as “country contributions”) were tabulated for each paper, aggregated across the full dataset, then ranked by the absolute number of contributions (Fig. 1). The vast majority of papers in MER have author affiliations in one of a few different Western countries. By contrast, only a very small percentage of total country contributions to MER come from Africa, South America, or Central Asia, and some countries in these world regions had no identified contributions to MER in 2015 or 2016.

To compare the geography of MER publication output with other fields, countries of affiliation were identified in large random samples of papers categorized under three other subject headings - “Education” (the parent term of “Education, Medical”), “Medicine”, and “Biological Science Disciplines”. For this portion of the study, all search parameters other than the MeSH terms themselves were identical to the search terms used for MER. To avoid double counting of papers, each paper was counted in the lowest income category represented among country contributions to the paper. In MER, a greater percentage of papers had authors based in high income countries and authors based in the US than in any other field. Moreover, MER had a higher proportion of authors from four English-speaking Western countries (the US, the UK, Canada, or Australia) than any other field evaluated (Table 1). The chi-square goodness-of-fit test was used to compare observations in each sample with expected values (the frequency in the MER population). For all pairwise comparisons of MER with other fields, the difference was statistically significant (*P* < 0.0001). The Gini-Simpson diversity (a quantitative measurement of diversity of a population) was also the lowest in MER of any field examined. Gini-Simpson diversity can vary from zero to one, with higher numbers indicating greater diversity in a population. These analyses indicate that MER, moreso than other fields, is dominated by authors from English-speaking Western countries.

**Table 1:** The Geography of Authorship in Different Fields of Medical Research. For each income category, the percentage of papers with authors from that category is shown. In cases of coauthorship, papers were counted in the lowest income category represented among authors. “US, UK, CAN, or AUS authors” indicates the percentage of total papers with authors from the US, the UK, Canada, or Australia. Gini-Simpson diversity was also calculated within each field. MER had the highest percentage of authors from high income countries and Western English-speaking countries and had the lowest geographic diversity of any field examined

	MeSH Term			
	Education, Medical	Education	Medicine	Biological Science Disciplines
High Income	89.5%	86.5%	85.8%	67.4%
Upper Middle Income	5.8%	8.7%	9.1%	25.1%
Lower Middle Income	3.8%	3.5%	4.0%	6.9%
Low Income	0.9%	1.3%	1.0%	0.6%
US, UK, CAN, or AUS authors	80.0%	74.0%	69.0%	48.0%
US authors	57.3%	52.3%	48.4%	36.7%
Gini-Simpson Diversity	0.753	0.802	0.855	0.905

**Fig. 2:**
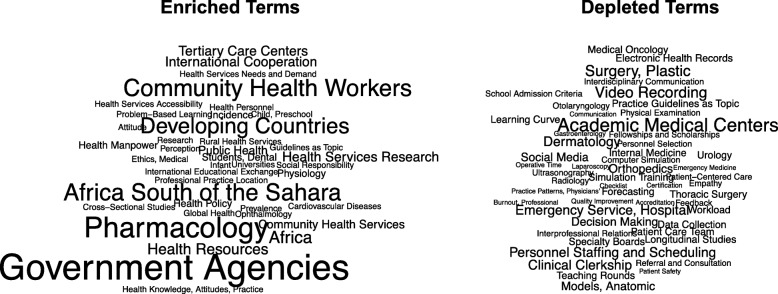
Differences in Topical Foci of Medical Education Research Papers by Income Status of Authors’ Countries The word clouds show MeSH terms that were > 2-fold enriched (left) or depleted (right) in papers that included authors based in low income or lower middle income countries. The size of each term is proportional to its over- or under-representation in papers from these countries

To assess whether the low geographic diversity in MER authorship could impact the research questions and topics pursued in the field, the enrichment of MeSH terms (relative to all MER papers) in publications with authors from low income and lower middle income countries was evaluated (Fig. 2). There were 39 enriched terms and 52 depleted terms using a two-fold cutoff. For clarity and readability, MeSH terms that are country names were excluded, but as expected, only low income or lower-middle income country names were enriched in papers with authors from these countries. To evaluate the possibility that the enrichment of terms was due to random noise, statistical significance of term enrichment was evaluated by simulation. The number of enriched terms was statistically significant as evaluated by 10,000 simulated samples of the same number of MER papers (*P <* 0.0001). Within the MER literature, terms associated with government, health delivery, health systems, and community health were enriched in papers with authors based in low income and lower middle income countries, while terms associated with specialty training, clinical practice and clinical teams, workplace concerns, and some teaching methods were under-represented in these papers.

## Discussion

Diversity can improve productivity in a variety of fields [11, 12], but there is limited research on geographic diversity in research and its impact on research questions. While it is unsurprising that papers authored in developing countries focus on locally-relevant research questions, this work indicates that MER stands to benefit from more research contributed by authors from developing countries. There are structural factors that make research in these settings more challenging [13, 14], and organizations that fund and support MER should work to counteract these factors. Researchers in low-resource settings may even face barriers in accessing the current literature, so every effort should be made to open up the benefits of MER research to the whole world. Although research costs may explain some geographic disparities in research output, MER research is not necessarily more expensive to conduct than research in other fields, such as Medicine and the Biological Sciences, which have greater geographic diversity of authorship. The recent origin of MER in wealthy Western countries [1] is a more likely explanation for geographic disparities in research output.

This work also identifies a number of MER topics that are not extensively studied in low income countries, particularly specialty training, hands-on training, clinical decision-making and practices, and concerns about workload, such as burnout and empathy. This analysis of the research literature points to possible gaps in medical education itself in developing countries. Future research could delve into the effects of international collaboration on the geography of MER publication output and the topics investigated in MER.

This analysis was restricted to papers from 2015 to 2016 published in English and indexed on PubMed. PubMed can include papers that do not meet certain editorial standards (through postings on PubMedCentral). By focusing on papers with certain MeSH terms, the study contains only papers from journals indexed by MEDLINE. This means that the journals included in this analysis meet certain editorial and quality standards [4, 15]. As a result, the study focuses more on a large population of *reputable* medical education papers, rather than *all* medical education papers. From 2015 onward, PubMed’s affiliation field can list affiliations of all authors, so this study improved over previous studies that only analyzed first author affiliations, but this does not guarantee that affiliation data is complete in PubMed. Moreover, with PubMed data it is not possible to determine whether authors are working in, and publishing papers in, countries other than their countries of origin. Although the computational method described here produced a low false discovery rate and high sensitivity, some affiliations were not geolocated, which could skew the results. This research relies on public data, rather than utilizing proprietary databases (such as Scopus or Web of Science), and all of the code for this work is open-source, so it should be straightforward for others to replicate and improve upon this work. There may be some divergence between a colloquial understanding of the term diversity and the quantitative measurements of diversity used in this paper. Notably, the calculations of diversity in this work only address diversity of countries of affiliation in different fields of research. Future studies should strive to evaluate other types of diversity in MER authorship.

## Conclusions

This analysis demonstrates that authors based in high income countries, particularly a few English-speaking Western countries, contribute the vast majority of research output in MER. Geographic disparities in research output are greater in MER than in any other field examined, and the quantitative diversity of authorship geography is lowest in MER. Research questions and topics of study vary considerably with the geography of authorship. Though authors from low income and lower middle income countries only contribute to a very small fraction of total papers in MER, the papers that they publish substantially increase the topical diversity of the MER literature.

## Data Availability

All of the data are publicly available on PubMed (https://www.ncbi.nlm.nih.gov/pubmed). The code used for this analysis is publicly available on GitHub (https://github.com/marshallthomas47/meded_research_landscape).

## Abbreviations

MER: Medical Education Research
MeSH: Medical Subject Headings
